# Post‐hatching development of the remote‐tactile bill‐tip organ in precocial shorebirds (Scolopacidae)

**DOI:** 10.1111/joa.70149

**Published:** 2026-04-07

**Authors:** Carla J. du Toit, Anyaise Green, Daniel J. Field

**Affiliations:** ^1^ Department of Earth Sciences University of Cambridge Cambridge UK; ^2^ Museum of Zoology University of Cambridge Cambridge UK

**Keywords:** bill‐tip organ, post‐hatching development, shorebirds, tactile foraging

## Abstract

Several groups of birds that rely on tactile foraging methods to locate prey exhibit specialized bill‐tip organs to sense tactile cues. Most scolopacid shorebirds (sandpipers and kin) employ the sensory capability of remote‐touch during foraging, allowing them to locate buried prey at a distance from their bills by detecting vibrational cues in the substrates they probe in. This is facilitated by a bill‐tip organ made up of dense clusters of mechanoreceptors housed within a constellation of neurovascular foramina in the bone at the tips of their bills. Bill‐tip organ morphology has been shown to correlate with foraging ecology; however, all previous research has focused on adult birds. Many tactile foraging birds, including scolopacids, have precocial young that forage independently within hours of hatching, yet remote‐touch foraging and its morphological correlates in juvenile birds remain unexplored. Here, we use μCT scans to describe the morphology of the bill tips of juvenile–adult pairs of several scolopacid species to characterize the post‐hatching development of the bill‐tip organ. We show that the osteological correlates of functional bill‐tip organs are present in juvenile scolopacids and describe changes in bill‐tip organ morphology during post‐hatching development. Our results suggest that juvenile scolopacids, and likely other precocial tactile foraging birds, are able to sense tactile cues with their bills from the point of hatching. Our data additionally suggest that previously described interspecific variation in bill‐tip organ morphology associated with taxon‐specific foraging behaviours and habitat preferences appears to develop after fledging and dispersal in scolopacids. These data reveal ontogenetic and interspecific differences in scolopacid bill‐tip organ morphology associated with differing ecologies and improve our understanding of foraging behaviour of juvenile shorebirds.

## Graphical abstract

Juvenile scolopacid shorebirds possess well‐developed osteological correlates of tactile bill‐tip organs, indicating sensory systems for remote‐touch foraging are present from early life. Ontogenetic changes in bill‐tip morphology suggest ecological specialisation and interspecific variation develop later, after fledging in these highly precocial shorebirds. Photo credits: Daniel Field and Kristof Zyskowski.

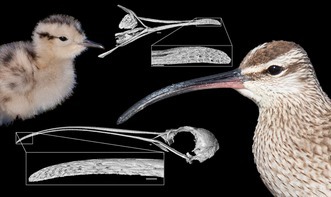

## 1 INTRODUCTION

Links between foraging ecology and avian bill morphology have long been the subject of ecomorphological investigation. Though well studied, most interspecific studies have focused on variation in adult bill morphology, with little regard to form‐function relationships in the bills of juvenile birds, despite the fact that the bills of many taxa undergo significant growth and geometric transformations during ontogeny (Arnaout et al., 2025; Brown, 1971; Cheng et al., 2025; Kushlan, 1975; Mallarino et al., 2011; Navalón et al., 2021; Thomson, 1994; Wu et al., 2006). Previous ecomorphological studies of juvenile bills have predominantly focused on how they are used in some taxa to signal adults for feeding; for instance, increased UV visibility of gape markers in cavity‐nesting birds guide parents to deposit prey in their bills (Hunt et al., 2003). This focus is relevant in altricial taxa, which rely on parents to locate and capture food items prior to fledging. However, juveniles of precocial taxa typically forage independently as soon as they leave the nest, often within hours of hatching. This foraging mode is typical of all palaeognaths (ostriches, tinamous and kin) and galloanserans (ducks, chickens and kin), as well as several taxa within the major clade Neoaves, including charadriiform shorebirds (e.g., plovers and sandpipers). Not all precocial chicks are equally independent with respect to locating their own food items (Ducatez & Field, 2021); for instance, some taxa traditionally considered precocial, such as oystercatchers (Charadriiformes: Haematopodidae), are fed by their parents for many weeks and only become fully independent closer to fledging (Norton‐Griffiths, 1969), while others are completely independent of any parental care from the point of hatching, such as megapodes (Galliformes: Megapodiidae) (Göth, 2002; Winkler et al., 2020c). Species that forage independently from a very young age may employ different strategies and exploit different food sources than their parents, even if they physically remain alongside adults for protection and/or temperature regulation via brooding, as in phalaropes (Charadriiformes: Scolopacidae: *Phalaropus*) (Mayfield, 1979). However, despite pronounced variation in foraging habits among juvenile precocial birds, potential ecological correlates of ontogenetic changes in bill morphology have yet to be investigated.

The importance of bill morphology is particularly pronounced in specialist foraging species. While the behavioural and gross morphological underpinnings of foraging specialisations are relatively well characterized [e.g., variation in bill and tongue size and shape, dietary specialisations in nectarivores, shorebirds and finches (Abbott et al., 1977; Barbosa & Moreno, 1999; Paton & Collins, 1989)], sensory specialisations also have potential to influence foraging strategy and may correlate with sensory organ morphology (Ausprey et al., 2026; du Toit et al., 2022; Thomas et al., 2006). Several precocial bird taxa are tactile foraging specialists, relying on their sense of touch to locate prey, either in combination with, or in the absence of, other sensory cues. For instance, many anseriform species (ducks, geese and kin) make use of dabbling and filter feeding to extract small food particles from water and highly saturated mud (Avilova et al., 2018; Gottschaldt & Lausmann, 1974). Kiwi (Apterygidae) and scolopacid shorebirds (sandpipers and kin) are able to locate prey at a distance from their bills by detecting vibrations from moving prey, or the changes in pressure gradients around hard‐bodied prey items, using a highly specialized sensory modality known as remote‐touch (Barbosa & Moreno, 1999; Bolze, 1968; Cunningham et al., 2007; Nebel et al., 2005; Piersma et al., 1998). All tactile specialists detect tactile cues via high densities of mechanoreceptors (predominantly Herbst and Grandry corpuscles) in the distal portion of their bills (Avilova, 2018; Bolze, 1968; Cunningham et al., 2007; du Toit et al., 2020a, 2022; Gottschaldt, 1985; Krogis, 1931). These tactile bill‐tip organs can be identified from osteological specimens, as their bill bones contain large numbers of neurovascular canals providing innervation for the organ via branches of the trigeminal nerve (Cunningham et al., 2013; Cunningham, Alley, et al., 2010; du Toit et al., 2020a, 2022; du Toit, Bond, et al., 2024; Nebel et al., 2005). These open to the external surface of the maxillary, premaxillary and mandibular bones, forming “sensory pits” that contain both nerves and the mechanoreceptors responsible for sensing tactile stimuli (du Toit et al., 2020a, 2022). The abundance and arrangement of mechanoreceptors and their associated pits differentiate bill‐tip organs from the condition observed in most birds, as almost all birds exhibit some neurovascular foramina and mechanoreceptors in their bills, albeit far fewer and much less densely packed, and with the receptors generally constrained to the dermis surrounding the bone (du Toit et al., 2020a). The number and arrangement of sensory pits in the bone have been previously established as reliable osteological correlates of the underlying soft tissue arrangement and associated sensory capabilities in broad phylogenetic studies across Neornithes, synthesizing data from soft tissues, osteological morphology and behavioural data (du Toit et al., 2020a; du Toit, Bond, et al., 2024).

Interspecific differences in bill‐tip organ morphology strongly correlate with various aspects of foraging ecology. In ducks (Anatidae), the numbers and density of both mechanoreceptors and the neurovascular canals are significantly higher in species that rely on tactile foraging compared to visual foragers (Avilova, 2018; Avilova et al., 2018). In ibises (Pelecaniformes: Threskiornithidae – altricial wading birds which also possess bill‐tip organs and utilize remote‐touch (Cunningham, Alley, et al., 2010; Cunningham, Castro, et al., 2010)), interspecific differences in remote‐touch bill‐tip organ morphology are strongly correlated with specific foraging behaviours and the physical properties of the substrates they forage in (du Toit, 2022; du Toit et al., 2022). However, existing comparative studies have focused exclusively on adult birds; as such, almost nothing is known about the ontogenetic development of the bill‐tip organ. In ducks, the mechanoreceptors of the bill‐tip organ are fully developed in late‐stage embryos and hatchlings and are found in the same arrangements as adults (Krogis, 1931; Soliman & Madkour, 2017). Yet, there have been no studies of the morphology of bill‐tip organs in juveniles or embryos of any other tactile specialist birds, nor investigations into ontogenetic changes in the structure of the bill associated with the neurovascular canals containing the nerves and vessels that innervate the sensory organ in any avian species.

Scolopacidae represent a particularly valuable clade in which to investigate the ontogenetic development and ecomorphology of bill‐tip organs. The clade is diverse, comprising 97 shorebird species and representing varied foraging behaviours and habitats, though most species rely to varying degrees on remote‐tactile foraging as adults (Angarita‐Báez & Carlos, 2023; Barbosa & Moreno, 1999; Nebel et al., 2005; Piersma et al., 1996). All adult scolopacids have bill‐tip organs (Bolze, 1968; du Toit et al., 2020a), but not all appear to employ remote‐touch foraging (e.g., phalaropes *Phalaropus* and turnstones *Arenaria*) (Jehl, 1988; Piersma et al., 1996; Whitfield, 1990). The presence of remote‐touch bill‐tip organs appears to be plesiomorphic in Scolopacidae and is likely vestigial in some species that do not undertake remote‐touch foraging (e.g., turnstones) (du Toit et al., 2020a). Some other Charadriiformes have long, thin bills used for tactile foraging, yet do not employ remote‐touch and lack a bill‐tip organ (e.g., oystercatchers *Haematopus*, stilts *Himantopus and* ibisbills *Ibidorhyncha*) (du Toit et al., 2020a), instead relying on direct contact with prey in addition to other sensory cues (e.g., vision) to locate prey (Hamilton, 1975; Hulscher, 1982; Ye et al., 2013).

All extant scolopacids are considered precocial, with their chicks leaving the nest hours after hatching, often as soon as their downy feathers have dried (Piersma et al., 1996). The degree of parental care after the chicks leave the nest varies among scolopacid taxa, with the parents of some species remaining near their chicks to signal danger, deter predators and/or aid in the chicks' temperature regulation via brooding (Piersma et al., 1996; Thomas & Székely, 2005). However, all scolopacid chicks are reported to forage completely independently of their parents as soon as they leave the nest (Piersma et al., 1996). Despite this, almost no data exist on the exact foraging behaviours exhibited by juvenile scolopacids prior to fledging. The primary reasons for this lack of data include the remote locations of breeding sites (most scolopacids breed in isolated Arctic and subarctic regions, usually very far from their non‐breeding habitats) (Piersma et al., 1996), and the difficulty in observing chicks foraging even in cases where they have been closely studied, due to their small size, highly cryptic plumage and tendency to hide and “disappear” into foliage when perceived predators including human observers are detected nearby (Mayfield, 1979). No previous studies exist on juvenile scolopacid tactile capabilities, or the development of their bill‐tip organs.

In this study, we describe the morphology of the osteological structures (neurovascular foramina) associated with remote‐touch bill‐tip organs in chick‐adult pairs of scolopacid shorebirds. The number and arrangement of these neurovascular foramina are well established as osteological correlates of tactile bill‐tip organs in Neornithes, differentiating them from the bills of other birds, which have far fewer and less densely arranged foramina in their bills (du Toit et al., 2020a; du Toit, Bond, et al., 2024). We provide the first comments on the presence and structure of bill‐tip organs in juvenile scolopacids and contextualise our results in terms of their implications for understanding the foraging and sensory ecology of juvenile shorebirds.

## 2 MATERIALS AND METHODS

### 2.1 Sample selection

Specimens were obtained from the University Museum of Zoology (University of Cambridge, UK). Due to the delicate nature of the highly pitted bones at the tip of the bill (particularly for juvenile specimens, which are smaller and not necessarily fully ossified yet), skeletal specimens are often not suitable for analysis as this region of the bone may be damaged when the rhamphotheca is removed. Thus, specimens prepared as round skins were examined, with CT scanning enabling visualization of the underlying bone. Each sample was first visually assessed for damage around the tip of the beak, which was then double‐checked once the scans had taken place. We included an adult and juvenile specimen of five scolopacid shorebird species known to possess bill‐tip organs at adulthood (Bolze, 1968; du Toit et al., 2020a): *Calidris minuta, C. ferruginea, Tringa erythropus, Numenius phaeopus* and *Phalaropus fulicarius* (specimen numbers are presented in Table S1, Supplementary Information). We also included a juvenile–adult pair of a non‐scolopacid shorebird, the oystercatcher *Haematopus ostralegus*, as a control species, as it is also a member of Charadriiformes with a long, thin bill at adulthood, but adults do not possess a bill‐tip organ and thus cannot employ remote‐touch foraging (du Toit et al., 2020a; Hulscher, 1982).

Exact ages of the juvenile samples varied, as we were limited by specimen availability. However, we selected juvenile specimens appearing to represent comparable stages of development: all juvenile specimens showed incomplete fusion of cranial sutures and incomplete ossification of their bones, were significantly smaller in absolute size compared to the adults and were still covered in downy feathers. Our sampling strategy followed similar studies into ontogenetic changes in avian cranial morphology (Plateau et al., 2024, 2026; Plateau & Foth, 2021).

Data from thirteen additional species of adult scolopacids were accessed from a previously published dataset (du Toit et al., 2020b) (species names and details included in Figure S2C), to provide a wider sample of scolopacids to compare to our five age‐paired species.

### 2.2 Data acquisition and analyses

The skulls of the specimens were scanned using a Nikon 49 Metrology XT H 225 ST high‐resolution CT scanner at the Cambridge Biotomography Centre at a resolution of 10 microns (due to the small size of the neurovascular foramina in the bone and the low density of juvenile bones). Segmentation and 3D surface mesh preparation of the premaxillary and mandibular bones were performed in *VGSTUDIO MAX* V2023.2.1 (Volume Graphics).

All measurements were taken from 3D surface meshes in *MeshLab* (Cignoni et al., 2008). Bill length was measured as the distance from the distalmost tip of the premaxilla to the craniofacial hinge. Using the “PickPoint” tool in *MeshLab*, the position of each neurovascular pit was selected at its rostral‐most point. The 3D coordinates of each pit were then used to calculate: the distance of each pit from the distal tip of the bill (which was selected and set as the origin, enabling trigonometric calculation of relative distance of each pit from the bill tip) and the nearest neighbour distance (NND) between pits, using the “nndist” function in the *spatstat.geom* package (Baddeley & Turner, 2005) in *R* (R Core Team, 2018). From this, we calculated the following variables for each specimen: total number of neurovascular pits; number of pits relative to bill length; maximum distance of pits from the tip of the bill; the proportion of the bill length pitted (max pit distance/bill length); and the average NND of pits on the bill.

Data from adult specimens of thirteen additional scolopacid species were sourced from a previously published dataset (du Toit et al., 2020b) to assess the total number of pits present and the average nearest neighbour distances between pits. These were originally taken from photographs of skulls, using similar methods to our approach, but using 2D instead of 3D coordinates for each pit. Linear regressions of the relationships among anatomical features were performed in *RStudio* (RStudio Team, 2016), using the *ggplot2, ggpubr* and *ggrepel* packages (Kassambara, 2023; Slowikowski et al., 2024; Villanueva & Chen, 2019).

## 3 RESULTS

**FIGURE 1:**
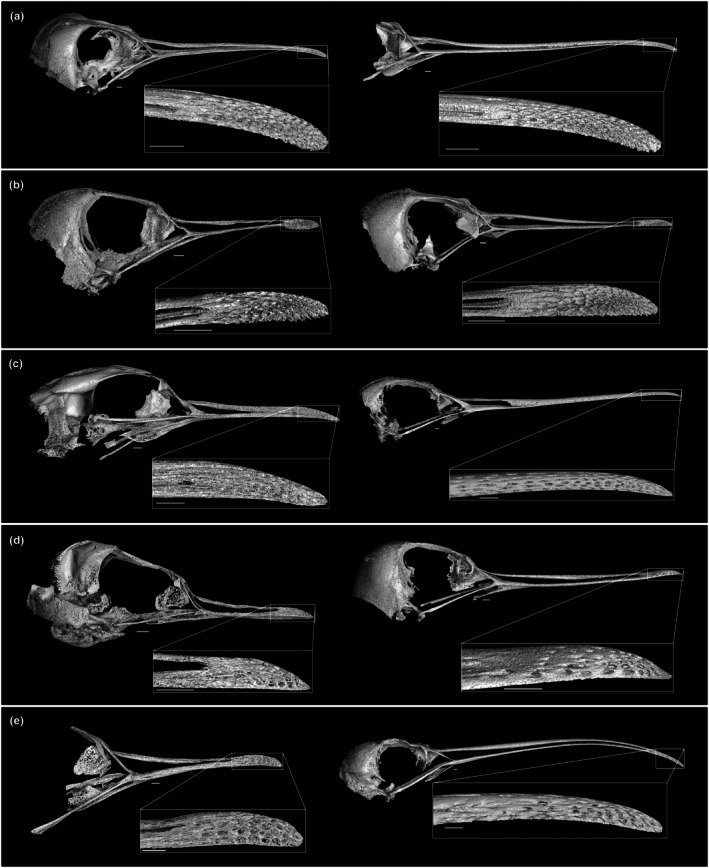
The skulls of five species of scolopacid shorebirds in lateral view taken from CT scans (juveniles on left, adults on right), with insets showing the tip of the bill to highlight the numerous neurovascular pits in the bone. (a) *Calidris ferruginea*; (b) *C. minuta*; (c) *Tringa erythropus*; (d) *Phalaropus fulicarius*; (e) *Numenius phaeopus*. Scale bars = 1 mm.

**FIGURE 2:**
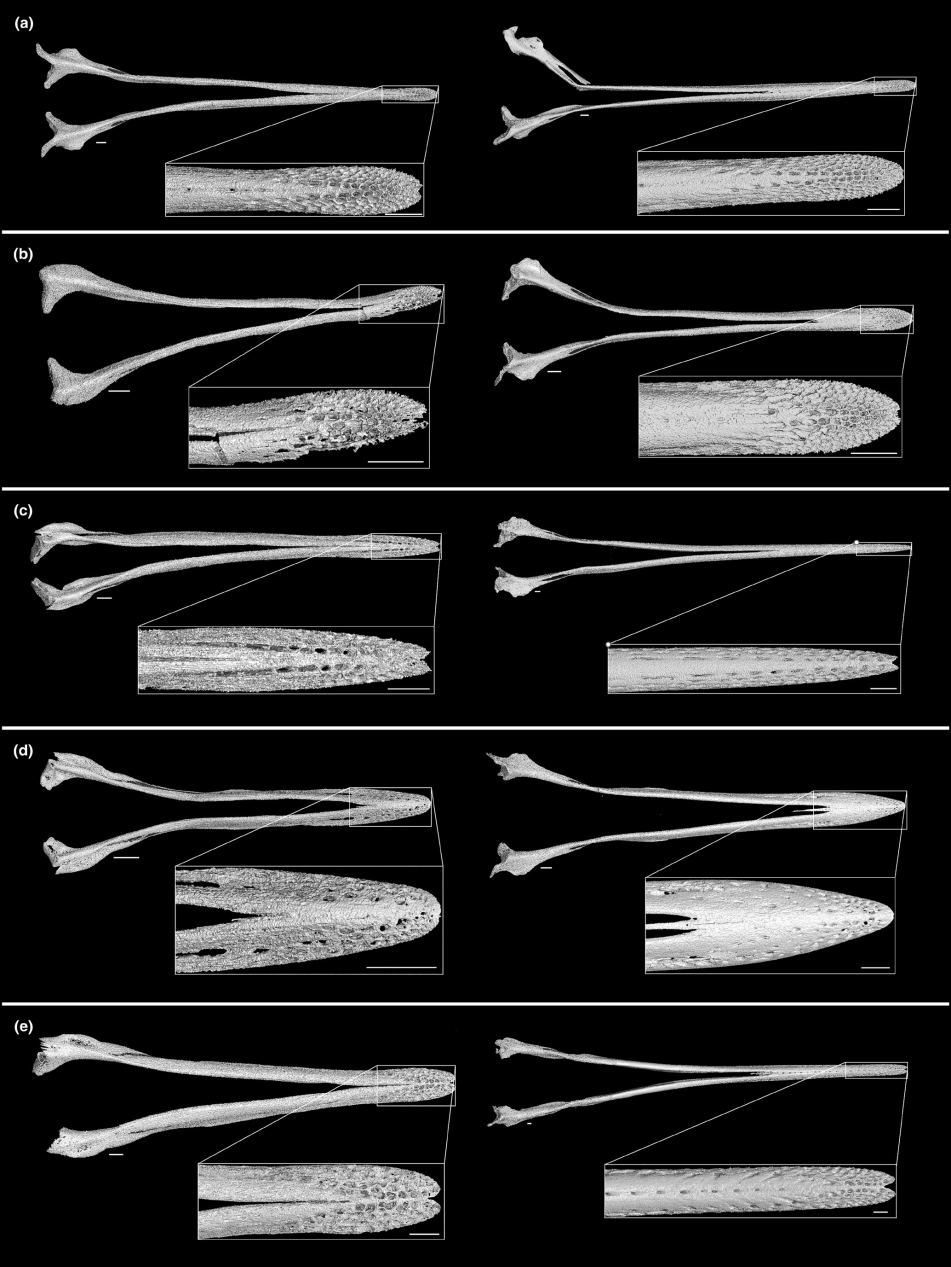
The mandibles of five species of scolopacid shorebirds in ventral view taken from CT scans (juveniles on left, adults on right), with insets showing the tip of the bill to highlight the numerous neurovascular pits in the bone. (a) *Calidris ferruginea*; (b) *C. minuta*; (c) *Tringa erythropus*; (d) *Phalaropus fulicarius*; (e) *Numenius phaeopus*. Scale bars = 1 mm.

Both adults and juveniles of all scolopacid taxa investigated exhibited neurovascular foramina on their bill bones (Figures 1, 2, and S1, Supporting Information). All adult and juvenile scolopacids show the same pattern of high densities of neurovascular foramina on the distal portion of their bills, characteristic of remote‐touch bill‐tip organs, which were not observed to the same degree in the oystercatcher *Haematopus ostralegus* at either age.

**FIGURE 3:**
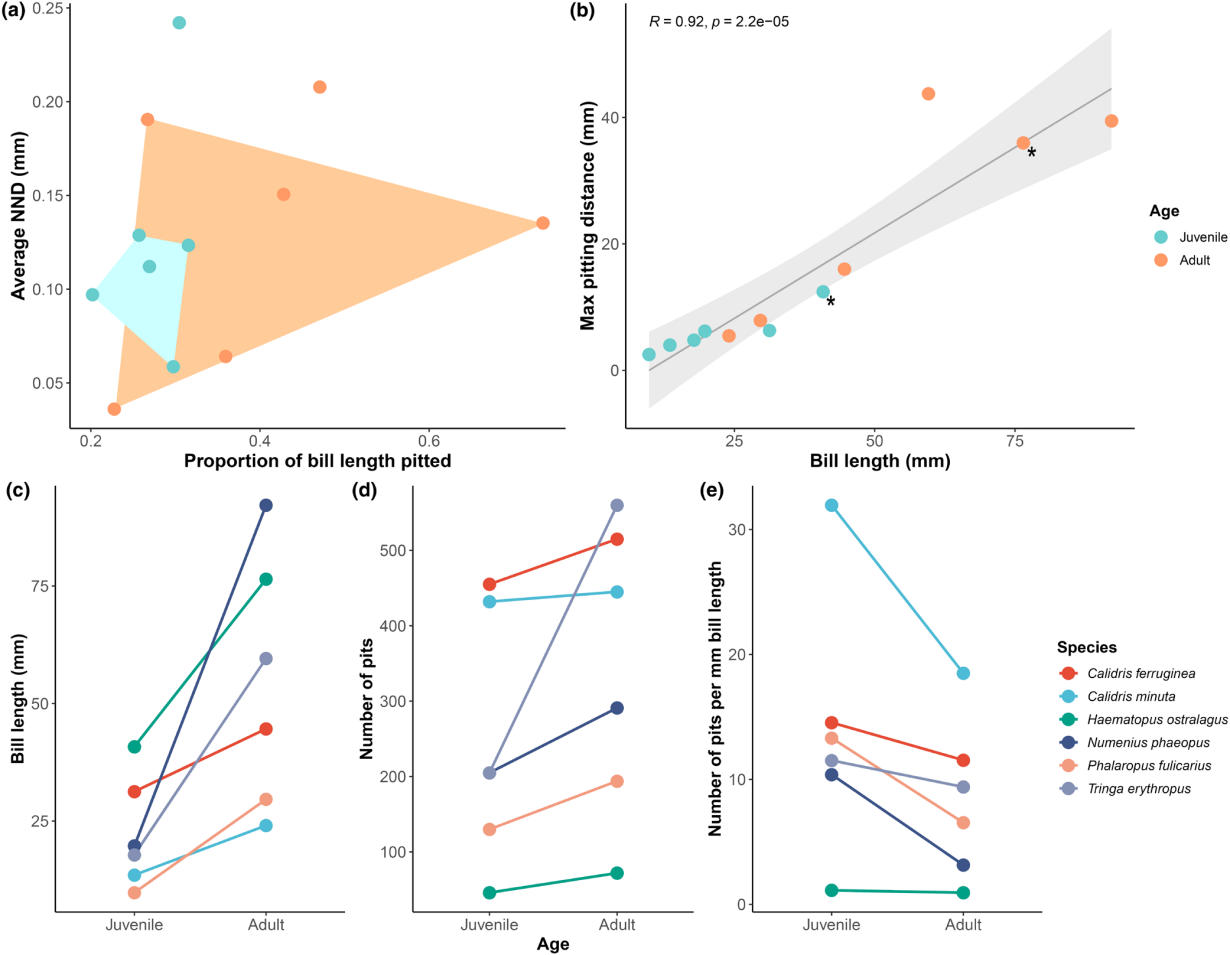
Ontogenetic changes in neurovascular pits in the bills of six species of Charadriiformes at different ages. (a) Changes in pitting patterns (proportion of bill length pitted vs. average nearest neighbour distance between pits) on bills in juvenile and adult birds: Juvenile scolopacid morphospace shaded in blue, adult scolopacid morphospace in orange. (b) Relationship between the length of the bill and the maximum distance of pits from the tip of the bill. Asterisks donate data from the non‐scolopacid charadriiform *Haematopus ostralegus*, which does not exhibit a bill‐tip organ. (c–e) changes in morphology between juvenile and adult birds, in: (c) bill length; (d) the total number of neurovascular pits on the bone; and (e) the number of pits per bill length (pits/mm).

Juvenile scolopacids show less interspecific variation than adults in the proportion of their bill length that is pitted and in the average distance between pits (see relative morphospaces in Figure 3a). The neurovascular canals in juveniles are constrained to the distalmost third (or less) of the bill length with pits situated very close together, while adults show more interspecific variation, with some species' bill‐tip organs occupying a much greater proportion of the bill length and with pits spread further apart than in juveniles. This is evident from images of the skulls (Figures 1 and 2), where the pits are spread further apart from each other and along the length of the bill in adults, with the most exaggerated ontogenetic changes seen in *Numenius phaeopus* (whimbrel) and *Tringa erythropus* (spotted redshank), while *Calidris minuta* (little stint) and *Phalaropus fulicarius* (red phalarope) show the least amount of change between adults and juveniles.

Across all species and age groups, the maximum distance of pits from the tip of the bill has a strong positive correlation to the length of the bill (Figure 3b). All juvenile bills are significantly shorter than adults (Figure 3c), with the greatest changes in bill length being found in the species, which have the longest bills at adulthood, namely: *N. phaeopus* (whimbrel), *T. erythropus* (redshank) and *H. ostralegus* (oystercatcher). All adults had greater numbers of neurovascular pits on their bills than did juveniles (Figure 3d), with a roughly 30% increase between juveniles and adults. The greatest increase in pit number was observed in *T. erythropus*, which exhibited more than double the number of pits in the adult. Despite the increase in bill length and total number of pits, the number of pits relative to bill length decreased in all adults (Figure 3e), with the greatest proportional change in *N. phaeopus* (70% decrease).

No significant correlation was observed between the number of pits and the pitted proportion of the bill length in scolopacids (Figure S2A). All six sampled scolopacids, including juveniles, exhibit very high numbers of pits closely spaced together. This was not observed in *H. ostralegus*, which does not exhibit a bill‐tip organ as evidenced by its osteological and soft tissue receptor arrangement, with far fewer, more sparsely arranged foramina (Figures S1 and S2b), and Herbst corpuscles being absent from the bill's distal tip and not located within the foramina (Heppleston, 1970). The arrangement in *H. ostralegus* matches the condition observed in the vast majority of extant birds, which lack bill‐tip organs (du Toit et al., 2020a). Only juvenile *Calidris* fall within the range of variation of adult remote‐touch foraging birds (Figure S2C, Supporting Information). Juveniles of the other three scolopacid species occupy a region of the morphospace comparable to those of non‐remote‐touch foraging scolopacid adults, such as *P. fulicarius* and *Arenaria interpres* (ruddy turnstone). The oystercatcher *Haematopus ostralegus* shows similar trends in terms of changes in sensory pit morphology between juveniles and adults to the scolopacids; however, the oystercatchers exhibit much lower pit numbers (both in terms of absolute number and relative to bill length; see Table S2) and greater average distances between pits at both ages than scolopacids (Figures 3a,d,e and S1).

## 4 DISCUSSION

### 4.1 Development of bill‐tip organs in juvenile scolopacids and implications for their foraging ecology

Juvenile scolopacid shorebirds have large numbers of well‐developed neurovascular foramina in the distal portions of their bill bones, in relatively similar numbers and arrangements to adults of the same species. This is the first evidence from any group of birds showing that the bone structures associated with tactile bill‐tip organs are well developed before adulthood. The distinctive arrangement and high numbers of sensory pits in juvenile scolopacids are clear when comparing them to the bills of juvenile oystercatchers, which lack bill‐tip organs (reflected by the relatively small numbers of sparsely arranged pits on their bill). Our data illustrate that neurovascular foramina associated with bill‐tip organs develop before adulthood (and indeed, before the bones of the skull are completely ossified) in all species sampled. The ability to identify tactile bill‐tip organs from the arrangement and number of neurovascular foramina in the bones in adult birds is well established (du Toit et al., 2020a; du Toit, Bond, et al., 2024). Our results thus indicate that juvenile osteological or fossil specimens may also be evaluated for the presence of bill‐tip organs, as the osteological correlates for the bill‐tip organ are already present at this life stage, though sampling of other juvenile tactile specialist taxa would provide better assurance of this.

Previous work on other precocial bird species (including other Charadriiformes) illustrates that mechanoreceptors known as Herbst corpuscles are already developed in late‐stage embryos and hatchlings, and in similar arrangements to those seen in adults, in both tactile and non‐tactile foragers (Gentle & Breward, 1986; Heppleston, 1970; Krogis, 1931; Soliman & Madkour, 2017). Only in one study of a highly altricial passerine species, the common blackbird (*Turdus merula*), were the receptors not fully formed at hatching (Pac & Malinovsky, 1988), and recent work has demonstrated pronounced differences in patterns of post‐hatching morphological development in other aspects of the avian feeding apparatus related to developmental mode (Plateau et al., 2026). Furthermore, a review of the development of Herbst corpuscles in the bill demonstrated that almost all species have receptors developed before hatching, and all precocial taxa sampled show complete development of these receptors in hatchlings, associated with the advanced degree of pre‐hatching development in precocial taxa (Saxod, 1996). Therefore, existing evidence suggests that these receptors are likely developed in precocial scolopacid juveniles, alongside their associated neurovascular foramina which we identify here. It would be ideal to confirm the presence of mechanoreceptors in the bills of juvenile scolopacids with soft tissue histological sections, though the availability of suitably fresh specimens for such destructive sampling may preclude such efforts. However, the neurovascular foramina in the bone form an integral part of the bill‐tip organ (containing nerves that innervate the organ and housing the mechanoreceptors in the distal portions of the foramina) and are strongly correlated with the presence and arrangement of soft tissue mechanoreceptors across various bird taxa, including scolopacids (du Toit et al., 2020a; du Toit, Bond, et al., 2024).

The presence of the osteological correlates of the bill‐tip organ in scolopacid chicks suggests that juveniles may be capable of remote‐touch foraging (Barbosa & Moreno, 1999; Nebel et al., 2005; Piersma et al., 1996). This could be highly beneficial for juvenile shorebirds, considering their precocial developmental mode and the fact that they forage independently as soon as they leave the nest, only hours after hatching (Piersma et al., 1996). Ducks, another precocial clade comprising numerous tactile foraging specialists that have been shown to develop bill‐tip organs before adulthood, are known to forage independently using tactile methods as juveniles (Pietz & Buhl, 1999). The ability to employ remote‐touch has been confirmed in adult birds (shorebirds, ibises and kiwi) using sensory assay experiments on captive birds (Cunningham, Castro, et al., 2010; du Toit, Chinsamy, & Cunningham, 2024; Gerritsen & Meiboom, 1985; Nebel et al., 2005; Piersma et al., 1998), and by analysing hypertrophy of the regions of the brain responsible for processing tactile information from the bill from soft tissue dissections of fresh brain material (Cunningham et al., 2013; Gutiérrez‐Ibáñez et al., 2010). Juveniles have yet to be included in such studies, but future work assessing this capability would be illuminating. Unfortunately, very little data exist on the foraging behaviour of juvenile scolopacids and future investigations on the topic are warranted (Piersma et al., 1996).

While the osteological correlates of the bill‐tip organ are already present in juvenile birds, there are differences in their morphology compared to the adults of the same species. In general, ontogenetic changes in the osteological component of the bill‐tip organ comprise a decrease in the proportion of total bill length that is pitted and an increase in the total number (and a decrease in density) of pits. These morphological differences between adults and juveniles appear to reflect an allometric increase in the number and spread of neurovascular foramina as the premaxilla and mandibular symphysis grow in length. Improved sampling of more species and a greater number of individuals per age group would help clarify the drivers of these changes in future studies. The proportion of the bill length exhibiting pitting has been investigated in adults of remote‐touch foraging ibises (Threskiornithidae) and has been shown to be positively correlated with the average depth of probe feeding (du Toit, 2022). This, in turn, is associated with probing in water and less compact substrates in various taxa, including scolopacids (Cunningham et al., 2007; Danufsky & Colwell, 2003; du Toit, 2022; Kelsey & Hassall, 1989). Thus, it could be that juvenile scolopacids, if they are able to employ remote‐touch, probe at shallower depths than adults (which is almost certainly true as a result of their shorter bills overall) and/or in drier substrates.

Illuminating the foraging behaviour of juvenile scolopacids is key to understanding their ecology and habitat requirements at a crucial point in their development. The first year of a bird's life, and indeed, the entire pre‐fledging period, are associated with the highest mortality rates, mainly due to increased risk of predation and starvation (Beauchamp, 2023; Lima, 2009). In precocial foraging chicks, such as scolopacids, starvation could result from their inability to locate their own prey. Lack of data on juvenile scolopacid foraging behaviour and requirements is problematic, as 25% of extant scolopacid species are globally threatened, including three that are critically endangered species (BirdLife International, 2024), and the complex factors influencing shorebird declines remain an active area of research (Chowdhury et al., in press). Indeed, in the last year, the Slender‐billed curlew (*Numenius tenuirostris*) became the first continental European bird species to be declared extinct (Buchanan et al., 2025). While our data on the morphology of their tactile organs suggests for the first time the reliance of juvenile shorebirds on this sensory system, further work is needed to investigate this in detail, and good behavioural data will be crucial for future ecomorphological studies.

### 4.2 Interspecific variation in ontogenetic trajectories

Interspecific variation in bill‐tip organ morphology (particularly in the distance between pits and the proportion of the bill occupied by the bill‐tip organ) is more exaggerated in adult scolopacids. Interspecific differences in bill‐tip organ morphology have been shown to be related to differences in foraging behaviour (e.g., reliance on remote‐touch) and the physical properties of the substrates the birds forage in (du Toit, 2022; du Toit et al., 2022). At adulthood, the five species investigated in this study use different foraging habitats outside their breeding ranges (the majority of the year), ranging from intertidal zones, mud flats, grasslands and even the open ocean in the case of phalaropes (Piersma et al., 1996). Likewise, they show variation in their diets and foraging behaviours within and between these different habitats. Whimbrels have a broad diet, often taking larger invertebrate prey (such as crabs) than many other scolopacids, and sometimes, foraging in fields and farmland away from water (Keel & Mallory, 2020; Turpie & Hockey, 1997). Curlew sandpipers have very generalized foraging strategies, ranging from highly tactile probing in wet mud, to visually gleaning prey off the sand and water surface (Mlodinow & Medrano, 2023; Puttick, 1979). Red phalaropes are exclusively visual foragers, and generally glean prey from the water, most frequently out to sea while swimming on the surface of the water (Mayfield, 1979; Tracy et al., 2020). Thus, the interspecific differences in bill‐tip organ morphology at adulthood likely reflect their differences in foraging ecology to some degree.

The lower interspecific variation in juvenile bill‐tip organ morphology could have several explanations. Changes in bill‐tip organ morphology are linked to the ontogenetic growth of the bill, as juveniles also show less variation in bill length than adults do. However, the number of pits and proportion of bill pitted are not strongly correlated with bill length in the species sampled. Additionally, juveniles of these species tend to forage in more similar habitats and are more similar to one another in terms of foraging ecology than adults are (Piersma et al., 1996). As discussed above, detailed data on juvenile foraging behaviour is limited. However, the majority of scolopacids breed in similar habitats (compared to the diversity of habitats utilized for foraging by non‐breeding adults). Namely, most breed within or close to the Arctic circle, in marshy freshwater wetlands. During the breeding period, adults tend to forage more similarly to each other than they do for the majority of the year, reflective of the similarities in their breeding habitats (Piersma et al., 1996). Therefore, as chicks must begin foraging several weeks before they can fly and disperse to more diverse foraging habitats, the relative similarity of their early foraging habitats provides evidence that they likely have more similar behavioural and sensory habits regarding prey location than do non‐breeding adults. Further, juveniles may be less reliant on remote‐touch foraging than adults, instead employing other senses to locate their prey, although additional data on the foraging ecology of juvenile shorebirds will be necessary to assess this hypothesis.

Scolopacids are typical among Charadriiformes in the suborder Scolopaci in being precocial with independently foraging juveniles (Baker‐Gabb et al., 2020; Gibson & Baker, 2012; Winkler et al., 2020a, 2020b, 2020d). However, unlike other representatives of this clade, the vast majority of scolopacids are long‐distance migrators, traveling between highly restricted breeding grounds and non‐breeding ranges characterized by vastly different habitats (Piersma et al., 1996). Whether the fact that scolopacids are the only members of this subclade to exhibit remote‐touch bill‐tip organs is related to their atypical breeding strategy remains to be investigated. Notably, both of these factors are likely related to highly precocial developmental modes—as adults do not remain long on the breeding grounds, chicks must forage independently from an early age, and the ability to utilize tactile foraging methods from a young age likely benefits these independent offspring. Disentangling the sequence by which these parameters evolved in future work may illuminate the selective drivers favouring the acquisition of some of the most distinctive aspects of scolopacid biology.

It would be worthwhile to compare these results from scolopacids to ontogenetic patterns in other remote‐touch foraging taxa. Kiwi are also precocial, so similar patterns of bill‐tip organ development in juveniles may be expected, if they are linked to juvenile foraging requirements. By contrast, ibises are altricial, and thus comparing them to scolopacids would be valuable to assess whether there are differences in the development of bill‐tip organs between altricial and precocial taxa. As neurovascular foramina are developed in the bones of the juvenile oystercatcher (which do not have bill‐tip organs), it is plausible that juvenile ibises also show some developed neurovasculature associated with bill‐tip organs, despite not having to forage for themselves until well after fledging. Notably, however, there are differences in the general morphology of the bill‐tip organs of adult scolopacids, kiwi and ibises, which are unlikely to be related to their developmental mode and rather may be related to their foraging behaviours and phylogeny (Cunningham, Alley, et al., 2010; du Toit, 2022; du Toit et al., 2020a, 2022).

## 5 CONCLUSIONS

The osteological correlates of bill‐tip organs associated with remote‐touch foraging are well developed in juvenile scolopacid shorebirds, indicating that these precocial, independently foraging offspring may be capable of tactile foraging from a very young age. This work provides the first evidence that the osteological structures associated with tactile bill‐tip organs are distinguishable in juvenile birds, and thus that these sensory organs can be identified in osteological and fossil specimens before adulthood. There are some differences between adult and juvenile bill‐tip organs, potentially correlated to ontogenetic niche partitioning in foraging ecology of these shorebirds. Adults show a greater degree of interspecific variation in bill‐tip organ morphology than juveniles, which likely reflects allometric growth of the organ, as well as possibly indicating that juveniles are specialised for more similar foraging habits than are adults, which employ a greater range of foraging behaviours outside the breeding season away from their breeding grounds. Future research into the evolutionary link between precociality and tactile foraging would be beneficial, both in scolopacids and other groups of birds. Our study also highlights the importance of addressing the lack of behavioural data on juvenile scolopacid shorebirds before fledging, and that this gap in our understanding of this unusual and threatened bird clade must be filled in order to facilitate further insights into their ecomorphology, evolution and conservation.

## AUTHOR CONTRIBUTIONS

CJDT and DJF conceptualized the project. CJDT designed data collection methods, prepared figures and wrote the manuscript draft. AG segmented CT scans. AG, DJF and CJDT collected and analysed data. CJDT and DJF provided supervision and oversaw the project. All authors were involved in editing the manuscript.

## CONFLICT OF INTEREST STATEMENT

The authors declare that they have no conflict of interest.

## Supporting information


Appendix S1.

## Data Availability

All data collected are included in the Supplementary Information accompanying this manuscript online. The 3D models generated from the CT scans of the specimens are publicly available online on *MorphoSource*: https://www.morphosource.org/projects/000757860?locale=en.
