# Supplementary material for: Post‐hatching development of the remote‐tactile bill‐tip organ in precocial shorebirds (Scolopacidae)
Source: J Anat. 2026 Apr 7;249(5):1087–98. doi: 10.1111/joa.70149 (PMC13398562; doi:10.1111/joa.70149)
Supplement: Supplementary file 1 [file JOA-249-1087-s001.docx]

Supporting Information for

**Post-hatching development of remote-tactile bill-tip organs in precocial shorebirds (Scolopacidae)**

Authors: CJ. du Toit, A. Green, & DJ. Field

Journal: *Journal of Anatomy*

**This PDF file includes:**

Figures S1 to S2

Tables S1 to S2

**Figure S1:** Images of the bones of the bill of Eurasian oystercatcher (*Haematopus ostralagus*) taken from CT scans at different ages, (a) juvenile and (b) adult. Top panels are lateral views of premaxilla and mandibles; bottom panels are ventral views of the mandibles; top right insets showing entire skull for scale. Scale bars = 1 mm

**Figure S2:** Data on neurovascular pits in the bones of the bills of Charadriiformes at different ages (adults shown in orange, juveniles in blue). A) Relationship between the proportion of the total bill length pitted (maximum distance of pits from the tip of the bill/bill length) and the total number of pits on the bill in five species of Scolopacid shorebirds. B-C) Measures used to differentiate birds with bill-tip organs from those without based on skeletal morphology in du Toit, *et al.* (2020a), namely the total number of pits vs. the average nearest neighbour distance (NND) between the pits Shaded region highlighting morphospace of adult birds known to rely on remote-touch foraging; dotted orange line identifying the morphospace of birds with bill-tip organs (based on morphology). B) Data from the six sampled Charadriiformes. C) Similar to B, but including data from an additional 13 adult scolopacid species [data from du Toit, *et al.* (2020b); all specimens from the Smithsonian National Museum of Natural History, Washington DC; see Table S2 in du Toit, *et al.* (2020a) for specimen accession numbers]. Grey dotted line showing species sampled in B.

**Table S1:** Museum accession numbers of specimens included in the study, all from the University Museum of Zoology (UMZC; University of Cambridge). All CT scans were taken from skin specimens, except for the adult *H. ostralagus*, which was from the osteological collection.

| **Species** | **Age group** | **Specimen number** |
| --- | --- | --- |
| *Calidris ferruginea* | Juvenile | UMZC 16/Sco/13/m/19 |
|  | Adult | UMZC 16/Sco/13/m/24 |
| *Calidris minuta* | Juvenile | UMZC 16/Sco/13/g/27 |
|  | Adult | UMZC 16/Sco/13/g/15 |
| *Numenius phaeopus* | Juvenile | UMZC 16/Sco/21/f/34 |
|  | Adult | UMZC 16/Sco/21/f/17 |
| *Phalaropus fulicarius* | Juvenile | UMZC 16/Pha/2/a/17 |
|  | Adult | UMZC 16/Pha/2/a/18 |
| *Tringa erythropus* | Juvenile | UMZC 16/Sco/27/a/4 |
|  | Adult | UMZC 16/Sco/27/a/2 |
| *Haematopus ostralagus* | Juvenile | UMZC 16/Hae/1/d/24 |
|  | Adult | UMZC 350 |

**Table S2:** Data collected on skeletal morphology of the bones of the bill from five species of Charadriiformes at different ages

| **Species** | **Age** | **Bill length (mm)** | **Number of pits** | **Average NND (mm)** | **Max pit distance from bill-tip (mm)** | **Number of pits per bill length (pits/mm)** | **Proportion of bill length pitted** |
| --- | --- | --- | --- | --- | --- | --- | --- |
| *Calidris ferruginea* | Juvenile | 31.27 | 455 | 0.10 | 6.32 | 14.55 | 0.20 |
|  | Adult | 44.61 | 515 | 0.06 | 16.03 | 11.54 | 0.36 |
| *Calidris minuta* | Juvenile | 13.52 | 432 | 0.06 | 4.02 | 31.95 | 0.30 |
|  | Adult | 24.05 | 445 | 0.04 | 5.48 | 18.50 | 0.23 |
| *Haematopus ostralagus* | Juvenile | 40.82 | 46 | 0.24 | 12.43 | 1.13 | 0.30 |
|  | Adult | 76.47 | 72 | 0.21 | 36.00 | 0.94 | 0.47 |
| *Numenius phaeopus* | Juvenile | 19.72 | 205 | 0.12 | 6.22 | 10.39 | 0.32 |
|  | Adult | 92.21 | 291 | 0.15 | 39.46 | 3.16 | 0.43 |
| *Phalaropus fulicarius* | Juvenile | 9.77 | 130 | 0.13 | 2.51 | 13.31 | 0.26 |
|  | Adult | 29.63 | 194 | 0.19 | 7.91 | 6.55 | 0.27 |
| *Tringa erythropus* | Juvenile | 17.79 | 205 | 0.11 | 4.79 | 11.52 | 0.27 |
|  | Adult | 59.59 | 560 | 0.14 | 43.78 | 9.40 | 0.73 |
